# Rare finding in peripheral nerve surgery: an unicentric Castleman disease presenting as median nerve tumour

**DOI:** 10.1080/23320885.2018.1525301

**Published:** 2018-10-25

**Authors:** Anne Carolus, Roland Schroers, Iris Tischoff, Kirsten Schmieder, Christopher Brenke

**Affiliations:** a Department of Neurosurgery, University Hospital Knappschaftskrankenhaus Bochum, Ruhr-University Bochum, Bochum, Germany;; b Department of Hematology and Oncology, University Hospital Knappschaftskrankenhaus Bochum, Ruhr-University Bochum, Bochum, Germany;; c Department of Pathology, University Hospital Bergmannsheil Bochum, Ruhr-University Bochum, Bochum, Germany

**Keywords:** Peripheral nerve tumour, unicentric Castleman disease, infiltrative growing tumour, surgery

## Abstract

A 51 year old man presented with progressive swelling in the upper arm. MRI revealed a solitary mass extending from the median nerve. Intraoperative finding was a tumour extending within the nerve in its proximal fibres. The histological result showed a Castleman disease.

## 1. Introduction

Tumours of the peripheral nerve system represent a broad and inhomogenous group. They can be subdivided into two categories according to their intrinsic or extrinsic growth: Peripheral nerve sheath tumors (PNST) and Peripheral non-neural sheath tumors (PNNST). Each entity includes benign and malignant tumours [1,2] (Table 1).

**Table 1.** Tumours of the peripheral nerve system.

MPNST	BPNST	MPNNST	BPNNST
MPNST	–schwannomas (= neurilemmomas/neuinomas) –neurofibromas	–metastasis (lung, breast, melanoma) –sarcomas –lymphomas	–ganglioncysts –hypertrophic neuropathy –lipomas –venous angiomas –hemangiopericytomas –hemangioblastomas –myositis ossificans –ostochondromas –ganglioneuromas –meningeomas –cystic hygromas –myoblastomas –granular cell tumors –epidermoid cysts –desmoids

MPNST: Malignant peripheral nerve sheath tumour; BPNST: Benign peripheral nerve sheath tumour; MPNNST: Malignant peripheral non neural sheath tumour; BPNNST: Benign peripheral non neural sheath tumour.

Diseases arising from lymphoid cells which are associated to the peripheral nerve system are extremely rare. A few cases of primary lymphoma [3], neurolymphomatosis [4] and lymph node metastasis [5] have been described previously. We present another entity of lymphoid tissue found in a peripheral nerve. It is known as Castleman diseaese (CM) or angiofollicular lymph node hyperplasia [6].

## 2. Case

A 51 year old man presented to our outpatient department with an increasing swelling in the right distal upper arm. He reported about local pain without radiation. The patient´s medical history was without previous infections, surgeries or other diseases. The mass in the arm presented solid and relocatable. The examination showed full strength in all upper extremity muscles, especially in the forearm flexors, in M. pronator, M. abductor pollicis brevis, M. flexor pollcis brevis, M. opponens pollcis and Mm. lumbricales I and II. No sensory loss in the upper arm, the forearm, the palm and dorsum of the hand and the fingers could be found.

**Figure 1.**
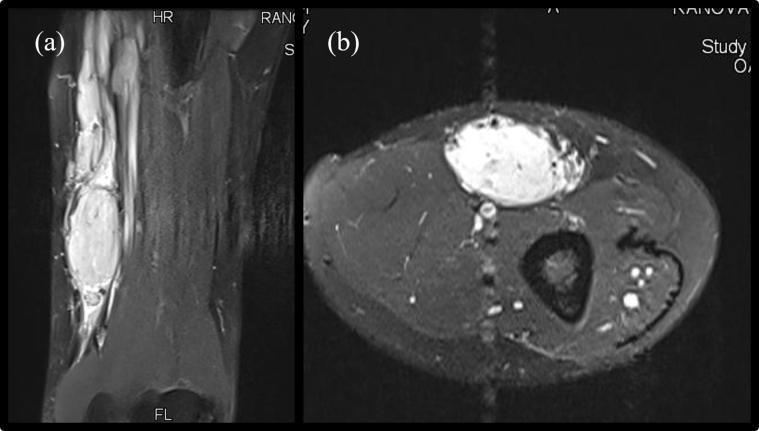
MRI of the upper arm showing a spindle shaped contrast enhancing mass in the median nerve course. (a) coronar view (b) axial view.

MRI of the upper arm showed a spindle-shaped homogeneously contrast enhancing mass. It was located some centimeters above the crook of the arm within the medial sulcus bicipitalis. In the imaging it showed a relationship to the median nerve main branch of the forearm or seemed to originate from part of its fibres, respectively. Its diameter was about 11 × 4 centimeters (Figure 1(a,b)). The primary diagnosis from the radiologist was schwannoma.

**Figure 2.**
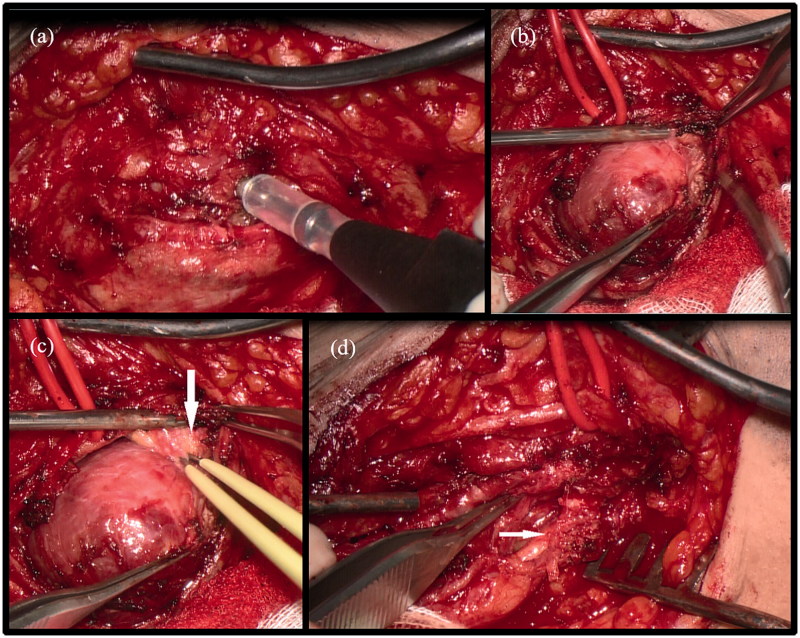
Intraoperative views of tumour dissection and removal. (a) Opening the capsule of the tumour and electric stimulation of fascicle-like structures. (b) Exposed tumour with a smooth surface in its middle part. (c) Exposed tumour, infiltrative growing in its proximal ending (arrow). (d) Tumorbed after removal of the median part, rest tumour embedding a nerve branch (arrow).

Surgical extirpation was indicated and performed. In its middle part the exposed tumour had a smooth capsule which was opened (Figure 2(a)). In its equator the surface had a good boundary to the surrounding tissue (Figure 2(b)). It did not extend to the muscles or tendons. In its distal and especially in its proximal ending the tumour showed a more infiltrative growth (Figure 2(c)). A feeding fascicle could be identified and was cut after ensuring by electric stimulation that it had no motor function. But with the intention to set no damage at the main nerve trunk approximately twenty percent residual tumour was left (Figure 2(d)).

The postoperative course was uneventful. The patient suffered a light hypesthesia in the forearm. This did not match to the supply territory of the median nerve which is the palmar hand. It rather corresponded to another skin nerve, possibly damaged by the approach. There was no new motor function deficit in the forearm flexors, in M. pronator, M. abductor pollicis brevis, M. flexor pollcis brevis, M. opponens pollcis and Mm. lumbricales I and II. A local upper arm pain vanished in the course of two weeks.

**Figure 3.**
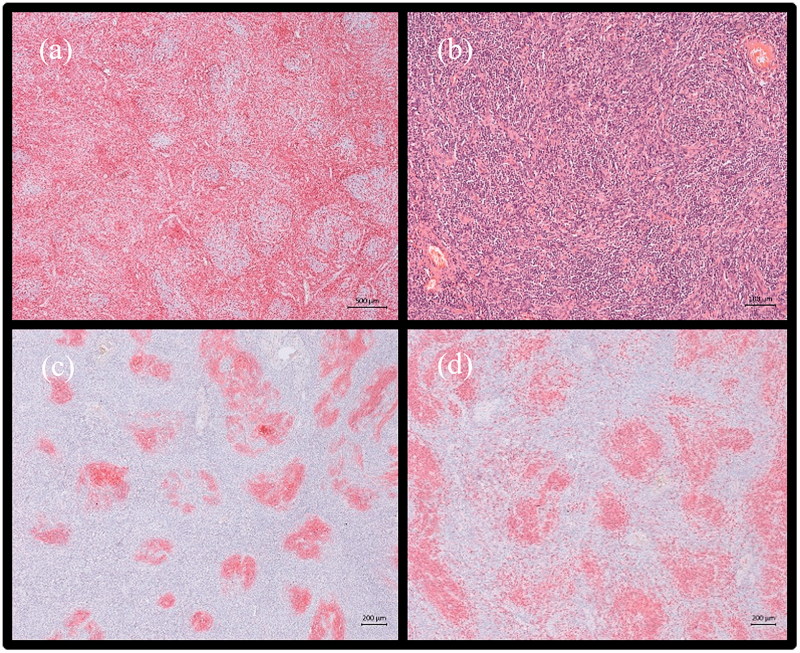
Histological stain sections. (a) Immunhistochemical staining for T-cells within the interfollicular zone. (b) In 10× HE stain section areas with high endothelial venules are demonstrated. (c) Immunhistochemical expression of CD20 demonstrating regressed germinal centers. (d) Immunhistochemical expression of CD23 shows extended network of follicular dendritic cells.

The final histological examination of the tumour showed typical criteria of the Castleman disease with an effaced architecture of a lymph node with regressed germinal centers and typical high endothelial venules (Figure 3(a,b)). Immunohistochemistry demonstrated regressed atrophic germinal centers (Figure 3(c)) and aberrant network of follicular dendritic cells (Figure 3(d)). The combination of these features ensured the diagnosis.

To exclude a multicentric disease the patient was admitted to the internal medical department. Entire virus tests including HIV were negative. A bone marrow biopsy showed a normal hematopoiesis without evidence for an infiltration by pathologic cells. A staging PET-CT showed no further organ manifestations. An unicentric form was approved in synopsis of all findings. In regard to the tumour rest and the curative approach of an unicentric M. Castleman the patient finally underwent a selective radiation of the upper arm [7].

In a 6-month follow-up, the partly sensory loss in the forearm had remained. Except for this, the patient had no nerve related problems or restrictions in everyday life except for the sensory loss in the forearm. In a 18-month follow up he reportet on full functionality of the arm. Currently, the area of the tumour is regularly examined with sonography.

## 3. Discussion

Retrospectively one can discuss what would have been the best neurosurgical management for our patient. Would he have benefit from a more radical tumour resection? Or contrariwise, would a frozen section have been helpful in such a case and a biopsy been the consequence? Should staging have had priority?

According to the literature unicentric Castleman disease has a good prognosis concerning the overall survival if treated by either complete surgical removal or a combination of surgery and radiation [7]. In this respect we think that an extirpation is superior to a sole biopsy in such a case. An even more radical approach with resection of a part of the nerve would be justified in cases where a rapid growth of the tumour is likely - in example as it is in MPNST. But in this case, being surprised by a complete new histological entity, there is a lack of long time experiences. We decided that peripheral nerve function preservation had priority, especially since a major nerve of the upper extremity was involved. Observation will show if this makes sense.

The histological dignity and growth behavior of a peripheral nerve tumour is irremissible information in this benefit-risk-assessment between complete removal on the one side and preservation of sensorimotor function on the other side. The features from Castleman disease distinguish clearly from the characteristic of tumours originating from the nerve sheath which is neoplastic proliferation of Schwann cell differentiation [8]. Nevertheless there is an increasing literature of MPNST mimics. Concerning the MRI finding and the infiltrative growth our case represents not a microscopic, but a macroscopic differential diagnosis of MPNST.

In our opinion the fact of its rareness, the challenging MRI, the malignant histological features, the necessity of interdisciplinary treatment and the lack of experience in the question of radicality justify that unicentric Castleman disease is registered in the itemization of peripheral nerve-associated malignant neoplasms.

## 4. Conclusion

Castleman disease is a group of lymphoproliferative disorders including special histological characteristics of lymph node. It is rare and may affect any body region. A manifestation at a solitary peripheral nerve has not been described so far. We present a case in which an unicentric Castleman disease mimicked a MPNST if dealing with the radiological and neurosurgical features. The pathological examination resolved the challenging case. An interdisciplinary treatment approach seems to be essential.
